# Recent Advances in CAR-Based Solid Tumor Immunotherapy

**DOI:** 10.3390/cells12121606

**Published:** 2023-06-11

**Authors:** Min Hwa Shin, Eunha Oh, Yunjeong Kim, Dae-Hwan Nam, So Young Jeon, Jin Hyuk Yu, Dohsik Minn

**Affiliations:** 1 Immune Research Institute, Seegene Medical Foundation, Seoul 04805, Republic of Korea; 2 Department of Diagnostic Immunology, Seegene Medical Foundation, Seoul 04805, Republic of Korea

**Keywords:** CAR-T, CAR-NK, CAR-M, cancer immunotherapy

## Abstract

Adoptive cell therapy using chimeric antigen receptor (CAR) technology is one of the most advanced engineering platforms for cancer immunotherapy. CAR-T cells have shown remarkable efficacy in the treatment of hematological malignancies. However, their limitations in solid tumors include an immunosuppressive tumor microenvironment (TME), insufficient tumor infiltration, toxicity, and the absence of tumor-specific antigens. Although recent advances in CAR-T cell design—such as the incorporation of co-stimulatory domains and the development of armored CAR-T cells—have shown promising results in treating solid tumors, there are still challenges that need to be addressed. To overcome these limitations, other immune cells, such as natural killer (NK) cells and macrophages (M), have been developed as attractive options for efficient cancer immunotherapy of solid tumors. CAR-NK cells exhibit substantial clinical improvements with "off-the-shelf" availability and low toxicity. CAR-M cells have promising therapeutic potential because macrophages can infiltrate the TME of solid tumors. Here, we review the recent advances and future perspectives associated with engineered immune cell-based cancer immunotherapies for solid tumors. We also summarize ongoing clinical trials investigating the safety and efficacy of engineered immune cells, such as CAR-T, CAR-NK, and CAR-M, for targeting solid tumors.

## 1. Introduction

**Figure 1:**
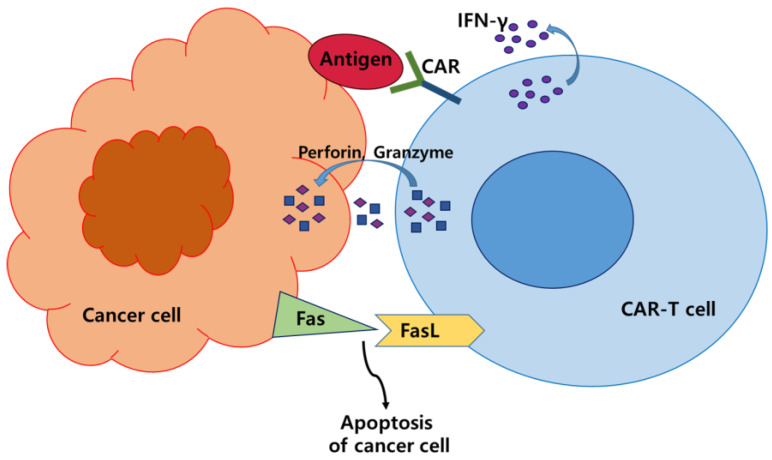
Schematic diagram of the CAR-T cell cytotoxic mechanisms against the cancer cells. Activated CAR-T cells can specifically recognize the tumor antigen and secrete perforin, granzyme, and IFN-γ to mediate anti-tumor activity. Death receptor pathway via Fas/Fas-L mediates the anti-tumor activity of CAR-T cells and leads to cancer cell apoptosis.

Owing to considerable side effects and resistance to traditional treatment options for cancer, including chemotherapy and radiation therapy, immunotherapy has emerged as a promising treatment option for various types of cancer by harnessing the ability of the immune system to target cancer cells [1]. Among these immunotherapeutic approaches, chimeric antigen receptor (CAR)-T cells have shown remarkable success in the treatment of hematological malignancies, including acute lymphoblastic leukemia, myeloma, and non-Hodgkin's lymphoma, and have received United States Food and Drug Administration (US FDA) approval for these indications [2]. CAR-T cells have direct anti-tumor activity against antigen-positive tumor cells by releasing perforin, granzyme, and Interferon-gamma (IFN-γ) [2] (Figure 1). Additionally, they mediate apoptosis via death receptor ligands such as FasL in antigen-negative tumor cells [2] (Figure 1).

The immunosuppressive tumor microenvironment (TME), limited tumor-specific antigens, tumor heterogeneity, cytokine release syndrome, and neurotoxicity restrict the use of CAR-T cells in solid tumors [3]. Alternative immune cells such as CAR-based natural killer (NK) cells and macrophages (M) have been shown to be effective strategies for overcoming barriers and targeting solid tumors [4,5]. The advantages, limitations, and potential strategies for improving the efficacy of CAR-T cell therapy are listed in Table 1. CAR-T cell therapy generally requires a single infusion [6] and shows strong efficacy and durable clinical responses [7,8] in hematologic malignancies [9,10,11]. The loss of antigen specificity and heterogeneity in solid tumors can be overcome by modifying CAR-T cells to target two or more antigens, such as bispecific CAR-T cells with two CARs with different individual single antigen recognition domains [12], Tandem CAR-T cells with two individual antigen recognition domains in one CAR [13,14], activating bystander effects by engineering CAR-T cells to express bispecific T cell engager (BiTE) to recruit and activate bystander T cells [15,16]. CAR-T cells designed to express chemokine receptors such as CCR4 and CCL22 and the CX3CR1-CX3CL1 axis [17,18,19,20], target stromal cell-associated antigens called fibroblast activation proteins (FAP) [21,22], and express immune checkpoint inhibitors [23,24] can overcome the difficulty of solid tumor trafficking and infiltration. The limited persistence of CAR-T cells in the immunosuppressive TME can be improved by activating cytokines—such as interleukin (IL)-12, IL-18, and IL-15 [25,26]—as well as modifying CAR-T cells to target immunosuppressive cells, including M2 macrophages, myeloid-derived suppressor cells (MDSCs), and regulatory T cells (Tregs) [27], conducting combination therapy with CAR-T cells and checkpoint inhibitors [28] or design CAR-T cells to express immune checkpoint inhibitors such as PD-1 and/or CTLA-4 monoclonal antibodies [23] to prevent exhaustion of CAR-T cells and overcome TME [23,28]. The toxicity of CAR-T cells can be reduced by inhibiting IL-6 [29] and adding a suicide gene, inducible caspase 9 (iCasp9), to the CAR construct to produce a homodimer with a chemical induction of dimerization (CID) drug (AP1903 or AP20187) for activated dimerization of caspase 9 and initiating apoptosis of CAR-T cells [30]. Additionally, "off-the-shelf" allogeneic CAR-T cells can reduce the risk of graft versus host disease (GvHD) [31] (Table 1).

The complex solid tumor microenvironment makes CAR-T cells less effective because it is difficult for them to infiltrate the tumors [32]. Researchers have explored the potential of CAR-T cell therapy for treating solid tumors by targeting antigens specific to solid tumors such as human epidermal growth factor receptor 2 (HER2) [33], mesothelin (MSLN) [34], and carcinoembryonic antigen (CEA) [35]. They have also modified CAR-T cells to better tolerate the tumor microenvironment by incorporating inhibitory receptors that can prevent T cell exhaustion or engineered CAR-T cells from secreting cytokines that can recruit other immune cells to the tumor site [36,37]. Clinical trials are underway to investigate the safety and efficacy of CAR-T cell therapy in various solid tumors, including lung [38], pancreatic [39], and ovarian [34]. Although the results thus far have been complex, there are promising signs of anti-tumor activity and improved survival rates in specific patient populations [40]. Although CAR-T therapy for solid tumors is still in the early stages of development, its potential benefits in treating solid tumors are notable, and ongoing research is focused on optimizing it to improve patient outcomes [40].

Recent advances in CAR-NK and CAR-M therapies have shown significant promise in overcoming these challenges and are now being investigated to expand the potential of immunotherapy to treat solid tumors [41,42]. CAR-NK therapy involves genetically modifying NK cells to express CAR, which targets specific cancer cell antigens. NK cells are part of the innate immune system and can target and kill cancer cells without prior antigen exposure [43], making them attractive candidates and promising alternatives to T cells for CAR therapy in solid tumors. CAR- NK therapy is still in the early stages of development; however, pre-clinical studies and early clinical trials have shown promising results in the treatment of various types of cancer including lymphoma, leukemia, and solid tumors [44]. One of the main advantages of CAR-NK therapy over CAR-T cell therapy is that NK cells are less likely to cause severe side effects such as cytokine release syndrome and neurotoxicity and do not require human leukocyte antigen (HLA) compatibility [4]. CAR-NK therapy has the potential to be a safe and effective treatment option for patients with cancer, and further research is needed to determine its long-term safety and efficacy [40].

CAR-M therapy is a promising approach involving genetically engineered macrophages, which are innate immune cells that engulf and destroy foreign or abnormal cells [45]. Similar to CAR-T and CAR-NK, CAR-M therapy involves modifying macrophages to express a CAR that recognizes and destroys cancer cells, as well as to modulate their function within the tumor microenvironment to promote an anti-tumor response [46]. CAR-M therapy is a promising immunotherapeutic approach for solid tumors, because macrophages are found in large numbers within the tumor microenvironment and play a key role in promoting tumor growth and metastasis [47]. Additionally, CAR-M cells can promote phagocytosis and tumor antigen presentation [48]. CAR-M therapy is still in its early stages of development, and several ongoing pre-clinical studies have investigated its efficacy in different types of solid tumors [47,49]. Clinical trials are currently underway to study the safety and efficacy of CAR-M therapy in various types of cancer, including solid tumors [40].

Despite promising pre-clinical and clinical results, several challenges need to be addressed to improve the efficacy of CAR-NK and CAR-M therapy in solid tumors, including improving their persistence and survival in the tumor microenvironment, enhancing their specificity and potency, and reducing off-target toxicity. This review aims to provide an overview of CAR-T cell therapy and address its limitations in solid tumors as well as its alternatives, such as CAR-NK and CAR-M, to circumvent these limitations. We also introduce ongoing clinical trials and recent advances in the development of CAR-based immune cells for solid tumor immunotherapy, including the challenges that still need to be addressed.

## 2. CAR-T Cell Therapy

The first US FDA-approved CAR-T cell therapy in 2017 was an anti-cluster of differentiation 19 (CD19) CAR-T named Kymriah, manufactured by Novartis, which targets B-cell malignancies [50]. Although this was a remarkable success, major limitations must be overcome, particularly for solid tumors. The major hurdles for CAR-T cells against solid tumors include limited persistence, poor trafficking, insufficient tumor infiltration, antigen escape, heterogeneity of tumor antigens due to immunosuppressive microenvironments, and life-threatening severe toxicities such as cytokine release syndrome (CRS) and immune effector cell-associated neurotoxicity syndrome (ICANS) [51]. CAR-T cells are also limited to trafficking, penetrating, and infiltrating solid tumors due to the physical tumor barriers, such as the tumor stroma [52,53]. Tumor stroma consists of stromal fibroblasts, endothelial cells, immune cells (macrophages and lymphocytes), and extracellular matrix (collagen, fibronectin, proteoglycans, hyaluronic acid) [54]. Myeloid-derived suppressor cells (MDSCs), tumor-associated macrophages (TAMs), and regulatory T cells (Tregs) infiltrate solid tumors and result in immunosuppressive TME [55]. These infiltrated immune cells and solid tumor cells release tumor-progressing cytokines (IL-6, TNF-α, TGF-β), chemokines (CCL2, CCL5, CCL18, CXCL8, CXCL12), and growth factors (EGF, PGF, IGF-1, IGF-2, PDGF) that promote tumor microenvironment immunosuppressive [56]. Poor T cell expansion and limited T cell persistence are the two main causes of unresponsive CAR-T cell therapy [57]. Additionally, T cell exhaustion derived from programmed cell death protein 1 (PD-1) or cytotoxic T-lymphocyte-associated protein 4 (CTLA-4) immune checkpoint pathways promotes the decrease in anti-tumor activity [58,59]. In combination with checkpoint inhibitors, CAR-T cells can overcome this limitation and produce potent anti-tumor activity [60,61]. Several approaches, including CAR-based engineering, have been used to overcome these limitations.

**Figure 2:**
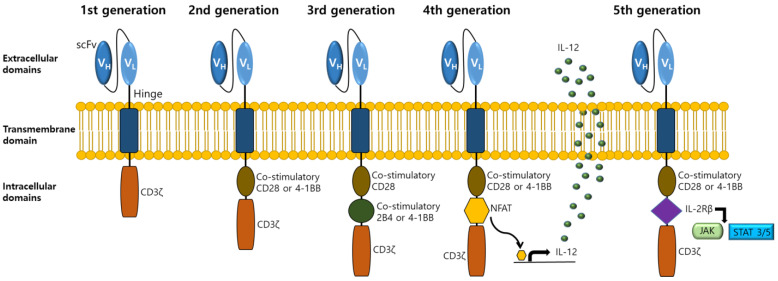
Progressive evolution of the CAR structure with modifications from the 1st to 5th generation. The central part of the CAR consists of three domains (extracellular, transmembrane, and intracellular domains). Components of the extracellular domain scFv region include heavy and light chains. Intracellular domains comprise CD3ζ signaling and co-stimulatory domains, such as CD28, 2B4, and 4-1BB. The fourth generation of CAR contains a nuclear factor of the activated T cells (NFAT) domain, which inducibly expresses cytokines such as IL-12. The fifth generation of CAR contains a JAK-STAT activation domain induced from IL-2Rβ.

The CAR construct was developed over five generations [7] (Figure 2). The main structure of the CAR is composed of extracellular antigen-binding, transmembrane, and intracellular signaling domains [7]. The extracellular antigen-binding domain is called a single-chain variable fragment (scFv) that targets tumor antigens [7]. The intracellular signaling domain consists of cytoplasmic tails of CD3z and one or two co-stimulatory molecules (CD28, 2B4, 4-1BB, etc.) correlated with CAR generation [7] (Figure 2). The fourth-generation CAR enhances killing activity by secreting cytokines such as IL-12 [25]. CAR-T cells engineered to express IL-12 could enhance killing activity by overcoming the immunosuppressive TME with increasing CAR-T cell persistence, proliferation, IFN-γ production, infiltration, and decreasing apoptosis, toxicity, and regulatory T cells in tumors [62,63]. The fifth-generation CAR has an intracellular domain of IL-2Rβ with a binding site for transcription factors, such as the Janus kinase (JAK) and signal transducer and activator of transcription 3/5 (STAT 3/5), to promote T cell proliferation and activation [64].

### 2.1. FDA-Approved CAR-T Cell Therapies

Since 2017, six CAR-T cell therapies have been approved by the FDA for the treatment of hematological malignancies [65,66]. They are Kymriah (tisagenlecleucel, CD19 CAR-T cells), Yescarta (axicabtagene ciloleucel, CD19 CAR-T cells), Tecartus (brexucabtagene autoleucel, CD19 CAR-T cells), Breyanzi (lisocabtagene maraleucel, CD19 CAR-T cells), Abecma (idecabtagene vicleucel, B-cell maturation antigen (BCMA) CAR-T cells), and Carvykti (ciltacabtagene autoleucel, BCMA CAR-T cells) [67]. Among these, four (Kymriah, Yescarta, Tecartus, and Breyanzi) are anti-CD19 CAR-T cells and two (Abecma and Carvykti) target BCMA. Table 2 shows the details of the approved indications, manufacturers, and dates of FDA approval of the six CAR-T cells. These six approved CAR-T cells are autologous, prepared from the patient by leukapheresis, and load a CAR targeting CD19 for B-cell malignancies and BCMA for multiple myeloma [67]. The CAR transgene was transduced into cells using a replication-incompetent retrovirus (Yescarta and Tecartus) or lentivirus (all others) and included a co-stimulatory molecule (CD28 for Yescarta and Tecartus; CD137, also known as 4-1BB, for all others) [7]. All were approved for treating hematological malignancies, including lymphomas, leukemia, and multiple myeloma (Table 2). To date, all approved CAR-T cell products share the adverse events of CRS, ICANS, cytopenia, and hypogammaglobulinemia [7]. Investigational approaches are currently focused on further potentiating the efficacy of CAR-T cells in non-responding patients and solid tumors

Clinical trials on FDA-approved CAR-T cell therapies in patients with hematological malignancies are ongoing, and some have been published [40,68]. The safety and efficacy of axicabtagene ciloleucel and tisagenlecleucel were investigated retrospectively outside the clinical trial [68]. Briefly, the overall response rate (ORR) was 60% (complete response (CR) 42% and partial response (PR) 18%) in the axicabtagene ciloleucel cohort. In the tisagenlecleucel group, ORR was 54% (34% CR and 19% PR) [68]. Both CRS and neurotoxicity were more frequent in the axicabtagene ciloleucel group (88% vs. 73%, *p* = 0.003, and 42% vs. 16%, *p* < 0.001, respectively) [68]. The 12-month progression-free survival (PFS) and overall survival (OS) for axicabtagene ciloleucel and tisagenlecleucel were 41% and 33% (*p* = 0.195), 51%, and 47% (*p* = 0.191), respectively [68]. This trial showed that the efficacies of axicabtagene ciloleucel and tisagenlecleucel were not significantly different.

NCT02435849, the global phase II ELIANA trial, tisagenlecleucel presents an overall remission rate of 81% in pediatric and young adult patients with relapsed or refractory B-cell acute lymphoblastic leukemia (R/R B-ALL) [40,69]. No significant long-term adverse events were reported [69]. Patients reported improved quality of life for up to 3 years [69]. The results of this trial suggest that tisagenlecleucel is a curative treatment option for devastating pediatric and young adult patients with R/R B-ALL [69]. The retrospective study of this trial in nonresponse or relapse after tisagenlecleucel demonstrated poor survival [70]. The OS at 12 months was 19% in nonresponders [70]. The OS at 12 months of CD19- relapse was significantly decreased to 30%, whereas CD19+ relapse was 68% [70]. These results provide poor survival in patients with nonresponse to tisagenlecleucel.

NCT02348216, a phase 2 of ZUMA-1 trial, investigated the safety and efficacy of axicabtagene ciloleucel after 5 years of follow-up in patients with refractory large B-cell lymphoma (LBCL) [40]. The objective response rate was 83% (58% CR). The median OS was 25.8 months, and the estimated 5-year OS rate was 42.6% [71]. No significant adverse events or deaths were observed [71]. These results provide the potential of axicabtagene ciloleucel in patients with aggressive B-cell lymphomas.

NCT03391466 is a phase 3, open-label, multicenter trial testing the efficacy of axicabtagene ciloleucel compared with standard care in patients with relapsed refractory DLBCL [40]. A response rate was 83% of the patients in the axicabtagene ciloleucel group and 50% of those in the standard care group (with a CR in 65% and 32%, respectively) [72]. Grade 3 or higher CRS occurred in 6% and grade 3 or higher neurotoxicities in 21% of the axicabtagene ciloleucel group [72]. These results provide evidence that axicabtagene ciloleucel therapy showed significant improvements compared with standard care in DLBCL.

### 2.2. Clinical Trials of CAR-T Cell Therapies in Solid Tumors

Recent ongoing representative clinical trials on CAR-T cells in patients with solid cancers are summarized in Table 3 [40]. These trials targeted tumor antigens such as MSLN, glypican-3 (GPC3), CEA, HER2, claudin18.2 (CLDN18.2), B4T2-001, CD70, natural killer group 2 member D ligand (NKG2DL), disialoganglioside (GD2), and mucin1 cell surface-associated C-Terminal (MUC1-C) [40] (Table 3).

NCT05693844 is investigating the effect of a novel CAR-T cell called CD40 ligand-expressing MSLN-CAR-T, targeting both MSLN antigen-expressing cells and CD40 ligand-expressing cells in advanced or metastatic solid tumors [40]. In a pre-clinical study, CD40 ligand-expressing MSLN-CAR-T cells showed a more potent anti-tumor effect than the previously reported CAR-MSLN T cells [40]. This trial will evaluate the safety and feasibility of CD40 ligand-expressing MSLN-CAR-T cell therapy [40].

NCT05621486 is studying the efficacy, tolerability, safety, and pharmacokinetics of autologous B4T2-001 CAR-T cells in patients with novel antigen BT-001-expressing advanced solid tumors [40]. B4T2-001 CAR-T cells are administered to patients after lymphodepleting chemotherapy using cyclophosphamide 300 mg/m^2^ and fludarabine 30 mg/m^2^ once daily for three consecutive days [40].

NCT05518253 is a single-center, double-arm, open-label phase I trial that evaluates the tolerability and safety of CAR-T cells in patients with CD70-expressing advanced/metastatic solid tumors, including renal cell carcinoma and ovarian and cervical cancer [40]. CD70 is a biomarker of renal cell carcinoma, non-small cell lung cancer, melanomas, and glioblastoma multiforme [73]. This trial consists of intravenous and intraperitoneal injection groups [40].

NCT05120271 is currently studying the safety and efficacy of BOXR1030 (a novel GPC3-targeted CAR-T cell that co-expresses glutamic-oxaloacetic transaminase 2 (GOT2)) after lymphodepletion (LD) chemotherapy with cyclophosphamide and fludarabine in subjects with GPC3-positive advanced solid tumors [40]. Pre-clinical data have shown that BOXR1030 demonstrates more powerful efficacy than GPC3-CAR alone under both in vitro and in vivo TME-like conditions [74].

NCT05382377 is a single-arm, single-center, open-label, early phase 1 study to investigate the safety and efficacy of autologous NKG2D CAR-T (KD-025) cells in the treatment of advanced NKG2DL-positive solid tumors [40]. NKG2DL is expressed at relatively low levels in normal tissues but is upregulated in hematologic malignancies and many types of solid tumors [75]. All patients in this trial underwent lymphodepletion chemotherapy before infusion with KD-025 [40].

NCT05239143 is a phase 1, open-label, multicenter, dose escalation, and expanded cohort study to evaluate the safety and efficacy of allogeneic P-MUC1C-ALLO1 CAR-T cells with Rimiducid administration in patients with advanced or metastatic epithelial solid tumors, including breast, ovarian, non-small cell lung, colorectal, pancreatic, renal cell, nasopharyngeal, head and neck squamous cell, and gastric cancers [40]. P-MUC1C-ALLO1 is an allogeneic CAR-T cell that targets cancer cells with aberrantly glycosylated MUC1-C antigens without targeting normal cells [76]. In a pre-clinical study, P-MUC1C-ALLO1 CAR-T cells showed the potential to treat multiple MUC1-positive solid tumors and in vivo efficacy against breast and ovarian cancer xenograft models [76].

NCT05199519 is an open-label, completed phase 1 trial to test the safety and efficacy of universal allogeneic IBI345 CAR-T cells in patients with CLDN18.2 antigen-positive solid tumors [40]. IBI345 has dual effects of Claudin18.2 recognizing antibodies and “modular” CAR-T cells to initiate anti-tumor activity [77]. The actual study completion date is 19 January 2023, and no results have been posted on ClinicalTrials.gov website [40].

NCT05103631 is evaluating the effect of autologous GPC3-CAR and IL-15 (CATCH T cells) in patients with GPC3-positive solid tumors [40]. The CATCH T cells will be infused into patients with GPC3-positive solid tumors after LD chemotherapy (Cytoxin and Fludarabine) [40]. In a pre-clinical study, GPC3-CAR and IL-15 showed enhanced anti-tumor activity in mouse xenograft models of GPC3+ solid tumors compared to GPC3-CAR alone [78].

NCT04581473 is an open, multicenter, phase 1b/2 trial investigating the safety and anti-tumor activity of CT041 autologous CAR-T cells targeting CLDN18.2 in patients with advanced gastric adenocarcinoma, pancreatic cancer, and gastroesophageal junction adenocarcinoma [40]. CLDN18.2, a gastric-specific isoform of the tight junction protein CLDN18, is a potential biomarker for the treatment of digestive system cancers [79]. One of three CT041 doses (2.5 × 10^8^, 3.75 × 10^8^, or 5.0 × 10^8^ cells) were given to 37 patients in the phase 1 trial interim report, and 94.6% of these patients showed grade 1 or 2 CRS [79]. The initial phase 1 results were promising, as the 6-month overall survival, overall response, and disease control rates were 81.2, 57.1, and 75.0%, respectively, in patients with gastric cancer [79]. Phase 1b of this trial was to determine MTD, DLT, and adverse events, and phase 2 to identify the efficacy and safety of CT041 autologous CAR-T cells versus the physician's choice of drugs (Paclitaxel, Irinotecan, Apatinib, or Anti-PD-1 antibody) as active comparators [40].

NCT04511871 is a single-arm, open-label, dose-escalation phase 1 study evaluating the safety and therapeutic tolerability of CCT303-406 CAR-modified autologous T cells (CCT303-406) in patients with relapsed or refractory stage IV metastatic HER2-positive solid tumors, including gastric, breast, and ovarian cancers and sarcomas [40]. Patients received LD followed by a single dose of intravenous CCT303-406 injection to determine dose-limiting toxicities (DLT) and maximum tolerated dose (MTD) [40].

NCT03170141 is a phase 1 trial to investigate the safety and efficacy of autologous GD2-specific fourth-generation safety-designed chimeric antigen receptor (4SCAR)-T cells in patients with GD2-positive glioblastoma (GBM) [40]. Four of the eight infused patients showed a partial response for 3 to 24 months [80]. The overall survival was 10 months after the infusion [80]. No severe adverse events were observed [80].

NCT04348643 is evaluating the efficacy, safety, infusion scheme, and recommended dose of CEA-targeted CAR-T cells in patients with relapsed/refractory CEA+ solid cancer [40]. CEA is a tumor biomarker expressed in more than 80% of patients with colorectal cancer [81]. The expression of CEA in normal tissue is low and limited to the cell membrane of digestive tract cells [81]. Its lack of expression in normal tissue cells makes CEA a powerful target for CAR-T cells.

NCT05812326 is a single-center, open-label, completed phase 1/2 trial evaluating the safety and efficacy of PD-1 gene knockout anti-MUC1 CAR-T cells (AJMUC1) in patients with advanced MUC1-positive breast cancer [40]. Three doses (3 × 10^5^ CAR T cells/kg, 1 × 10^6^ CAR T cells/kg, and 3 × 10^6^ CAR T cells/kg) with one, two, three, or more cycles of infusions are set [40]. The actual study completion date is 16 November 2022, and no study results have been posted on ClinicalTrials.gov website [40].

NCT03373097 consists of phase I, the dose escalation phase, and phase II, the expansion phase, to test the safety and feasibility of autologous GD2-CAR-T cells for treating pediatric or young adult patients with high-risk and/or relapsed/refractory neuroblastoma [40,82]. GD2-CAR-T cells are effective against glioblastoma lines and patient-derived cells in vitro and in vivo [83]. Patients are infused with 1.0–10.0 × 10⁶/kg GD2-CAR-T as a single dose after lymphodepletion with conventional chemotherapeutic agents [40]. GD2-CAR-T cells have a suicide gene (inducible Caspase 9) controlling the apoptotic pathway of infused GD2-CAR-T cells to prevent relevant toxicities [40]. Related to this trial, another study demonstrated that polymorphonuclear-MDSC attenuated the effect of GD2-CAR-T cells in patients with neuroblastoma [84].

NCT03198546 is a phase 1 trial testing the safety and efficacy of anti-GPC3-7 × 19 CAR-T cells (GPC3 CAR-T cells to secrete human IL-7 and CCL19) in patients with advanced hepatocellular carcinoma (HCC) expressing GPC3 [40]. After 30 days of post intratumor injection of anti-GPC3-7 × 19 CAR-T, patients with advanced HCC experienced complete tumor disappearance [85]. These results demonstrated that anti-GPC3-7 × 19 CAR-T cell therapy provides improved efficacy against solid tumors.

NCT03851146 is a phase 1, open-label, single-center, completed trial evaluating the safety and efficacy of a single infusion of LeY CAR-T cells in patients with Lewis Y antigen expressing advanced solid tumors [40]. LeY CAR-T cells are engineered from autologous peripheral blood T cells [40]. LeY is a difucosylated carbohydrate antigen expressed at a high copy number on various solid tumors, including ovarian, lung, breast, and colon cancer [86]. LeY CAR-T cells secrete enhanced levels of IFN-γ, TNFα, and IL-2 with direct stimulation through LeY-expressing tumor targets [87]. The actual study completion date is 1 April 2022, and no study results have been posted on the ClinicalTrials.gov website [40].

NCT02761915 is a completed phase 1 study to test the safety and feasibility of autologous 1RG-CAR-T (anti-GD2 CAR-T cells) for patients with relapsed or refractory neuroblastoma [40]. GD2 is a marker of the surface of neuroblastoma cells [88]. Two out of the six patients who received GD2 CAR-T cells experienced grade 2 to 3 CRS, and three showed regression of soft tissue [88]. The results of this trial demonstrated that GD2 CAR-T cells are feasible and safe but need more modification of CAR to have better efficacy.

## 3. CAR-NK Cell Therapy

### 3.1. NK Cells, a Potential Alternative for CAR-Based Solid Tumor Immunotherapy

NK cells are CD3-negative and CD56-positive innate immune cells that constitute 5~15% of peripheral blood mononuclear cells (PBMC) in humans [43]. They act as effector cells and are the first line of defense against tumors and viral infections without prior memory and are independent of tumor antigens [89]. They also regulate cancer cell death by patterned recognition of target ligands [90,91]. CAR-T cells can only target tumor cells using specific antibodies against scFv, whereas NK cells can be widely activated through their activating and inhibitory receptors [92]. The balance between activating and inhibitory receptors in NK cells is important for regulating their cytotoxic activity. The activating receptors of NK cells, such as DNAX accessory molecule-1 (DNAM-1), NKG2D, NKp46, NKp44, and NKp30, lead NK cells to kill tumor cells by secreting perforin and granzyme B [43]. Activated NK cells can induce target cell death via the death receptor pathway [93]. The death ligand expressed on the surface of NK cells binds to the death receptor on target cancer cells and promotes cancer cell death. NK cells express death ligands such as Fas ligand (FasL) and/or TRAIL [94,95]. FasL, a type II transmembrane protein, is expressed on the surface of NK cells and interacts with Fas [96]. When FasL binds to Fas, it activates the target cells' apoptosis signaling and leads cancer cells to death [96]. TRAIL is also a transmembrane protein type II and regulates the immune system's response [97]. TRAIL can bind to TRAIL receptors (DcR1(TRAIL-R3), DcR2(TRAIL-R4), OPG, DR4 (TRAIL-R1) and DR5 (TRAIL-R2)), and induce apoptosis of target cancer cells [98].

The interaction between killer cell Ig-like receptors(KIRs) and major histocompatibility complex class I promotes the inhibition of NK cell activation [43]. NK cells are also involved in antibody-dependent cellular cytotoxicity (ADCC) [43,99].

CAR-NK cells can be generated from various sources, including peripheral and cord blood, induced pluripotent stem cells (iPSCs), and cell lines [99,100,101,102,103]. The tumor-killing activity of CAR-NK cells can be regulated by CAR-dependent and -independent ADCC mechanisms [99]. CAR-NK cells are safer and have a lower risk of CRS, neurotoxicity, and GvHD than CAR-T cells; therefore, "off-the-shelf" allogeneic therapy is available with CAR-NK cells [99].

Although CAR-NK cells have shown promising advantages over CAR-T cells, substantial limitations and obstacles remain. CAR-NK cells have difficulty infiltrating solid tumors because of the immunosuppressive TME and tumor heterogeneity [43]. To enhance tumor infiltration of CAR-NK cells, researchers have modified them to express chemokine receptors and target immunosuppressive factors in TME such as Tregs, mesenchymal stromal cells, and cancer-associated fibroblasts [41,104]. Since the genetic modification efficiency of NK cells by viral transduction is generally very poor, researchers have developed effective methods using non-viral electroporation (mRNA, transposon) and enhancers of viral transduction (retronectin, vectofusin-1, and PEG2) [105,106,107,108]. NK cells generally require feeder cells for ex vivo expansion to boost their proliferation [43]. Engineering feeder cells expressing immunostimulatory cytokines and signaling molecules such as IL-21, IL-15, and 4-1BB can significantly improve the efficiency of the ex vivo expansion of CAR-NK cells [109,110]. Additionally, engineering CAR-NK cells to express IL-12, IL-15, and IL-18 can enhance their persistence and durability [104,111,112]. This approach has been applied to produce memory-like CD19-CAR-NK cells containing IL-12, IL-15, and IL-18 [111]. The anti-tumor activity of these cells against B-cell lymphoma was significantly elevated in vitro and in vivo compared with that of CD19-CAR-NK cells alone [111]. A phase I/IIa clinical trial (NCT03056339) of IL-15-expressing cord blood-derived CD19-CAR-NK cells for the treatment of relapsed or refractory CD19-positive non-Hodgkin's lymphoma or chronic lymphocytic leukemia was conducted and completed at the University of Texas MD Anderson Cancer Center [40,112]. In this trial, the infusion of CD19-CAR-NK cells was unrelated to CRS, GvHD, neurotoxicity, or secretion of inflammatory cytokines, with controllable safety issues [112]. Eight of 11 CD19-CAR-NK-treated patients showed a response, and of these, four with lymphoma and three with chronic lymphocytic leukemia showed complete remission [112]. The immunosuppressive TME of solid tumors reduces the persistence of NK cells and inactivates their killing activity by upregulating the checkpoint molecules PD-1, PD-L1, and immunosuppressive factors such as transforming growth factor-beta [113]. CAR-NK cells designed to secrete immune cell-stimulating cytokines such as IL-12, IL-18, and IL-15 can enhance the persistence of CAR-NK cells in the TME of solid tumors [41,106,114]. Additionally, combination therapy with immune checkpoint inhibitors can prevent the inactivation of CAR-NK cells in the immunosuppressive TME [41,104]. Because engineered NK cells can avoid GvHD and CRS and have shorter lifespans than other immune cells [115,116], CAR-NK cells seem safer than CAR-T cells for the clinical application of many types of solid tumors [44]. Table 4 summarizes the advantages, limitations, and potential strategies for improving the efficacy of CAR-NK cell therapies.

### 3.2. Clinical Trials of CAR-NK Cell Therapies in Solid Tumors

Ongoing clinical trials of CAR-NK cells in patients with solid cancers are presented in Table 5 [40]. NCT05528341 is a phase 1, open-label study evaluating the safety and efficacy of NKG2D-CAR-NK92 cells in treating patients with relapsed or refractory solid tumors [40]. NKG2D-CAR-NK92 cells were infused intravenously twice a week at a starting dose of 0.5 × 10^6^–2 × 10^6^/kg [40]. After three weeks of infusion, the efficacy is evaluated [40].

NCT05410717 is a phase1/2, open-label, single-arm trial investigating the safety and feasibility of Claudin 6 (CLDN6) targeting autologous CAR-NK cells in patients with CLDN6-expressing advanced solid tumors, including ovarian and testicular cancers [40]. Autologous CLDN6-CAR-NK cells were engineered to express CCL19/IL7 and scFv targeting PD-1/CTLA-4/Lag-3 to increase the anti-tumor efficacy against CLDN6-positive solid tumors [40].

NCT05194709 is a single-arm, open-label, early phase 1 clinical trial evaluating the safety, efficacy, and tolerability of anti-5T4 (oncofetal trophoblast glycoprotein) CAR-NK cells for treating patients with advanced solid cancers [40]. 5T4 is a 72-kDa transmembrane oncofetal antigen shared between human trophoblasts and cancer cells [117] and is expressed in advanced human solid tumors, including ovarian, prostate, colorectal, and renal cancers [117]. However, its expression is limited to normal tissue [117]. A pre-clinical study showed that autologous anti-5T4 CAR-T cells effectively target 5T4-expressing autologous tumors in an in vitro co-culture and an in vivo mouse xenograft model of ovarian cancer [117]. Another pre-clinical study demonstrated that anti-5T4 CAR-cytokine-induced killer cells efficiently target 5T4-expressing nasopharyngeal carcinoma cells in vitro [118].

NCT05137275 is an open-label, dose-escalation and extension, early phase 1 study investigating the safety, tolerability, and efficacy of conjugated antibody-based ready-to-use allogeneic CAR-NK (CAR-raNK) cells targeting 5T4 for treating patients with 5T4-expressing locally advanced or metastatic solid tumors, including non-small cell lung, breast, and colorectal cancers, and mesothelioma [40].

NCT03940820 phase 1/2 study testing the safety and efficacy of anti-ROBO1 CAR-NK cells in patients with solid tumors [40]. ROBO1 has been shown to be expressed in various solid tumors, including pancreatic cancer [40]. The pre-clinical study showed a significant decrease in tumor size and volume in the anti-ROBO1 CAR-NK-treated orthotopic mouse model of pancreatic cancer [119]. The estimated study completion date is May 2022, and the status is posted as unknown on the ClinicalTrials.gov website [40].

NCT 03,415,100 is a pilot phase 1 study testing the safety and feasibility of autologous and allogeneic anti-NKG2DL CAR-NK cells in patients with metastatic solid tumors [40]. Three patients are infused with intraperitoneal or intratumoral injections of anti-NKG2DL CAR-NK cells [40]. Pre-clinical results showed that NKG2D-CAR-NK cells showed a significant anti-tumor effect against HCT116 colorectal cancer cells in vitro and in vivo mouse xenograft models [120]. The estimated study completion date is December 2019, and the status is posted as unknown on the ClinicalTrials.gov website [40].

NCT02839954 is a phase 1/2 trial investigating the safety and efficacy of anti-MUC1 CAR-NK cells in patients with relapsed or refractory solid tumors, including GBM, HCC, breast, pancreatic, gastric, and colorectal cancer expressing MUC1 [40]. This trial is based on an estimated study completion date of July 2018, and the status is posted as unknown on the ClinigalTrials.gov website [40].

## 4. CAR-Macrophages

### 4.1. Macrophage, a Potential Alternative for CAR-Based Solid Tumor Immunotherapy

CAR-T cell therapy has shown efficacy in hematologic malignancies, but its application in solid tumors has proven challenging because T cells have a limited potential to infiltrate and survive in the TME [46]. To circumvent these limitations, researchers have explored macrophages as promising candidates for the next CAR platform because of their capacity to be the most abundant and highly infiltrated into the solid tumor TME [121]. Macrophages are one of the main components of the innate immune response and attack and kill both infected and abnormal cells [121]. They also exhibit phagocytic activity against tumors and present antigens to T cells to activate adaptive immunity [47]. Moreover, macrophages secrete cytokines and chemokines to modulate and remodel the solid tumor immunosuppressive TME [47]. Based on their phenotype and functional characteristics, macrophages are categorized into M1 and M2 macrophages [122]. The molecular markers for M1 in humans are CD36, CD80, CD86, MHC-II, inducible nitric oxide synthase (iNOS), interferon regulatory factor 5 (IRF5), and STAT1, and for M2 in humans are CD163, CD206, CXCR1, CXCR2, Dectin-1, Arg1, IRF4 and STAT6 [123,124]. M1 macrophages regulate pro-inflammatory roles in the TME and play a major role in anti-tumor activity, whereas M2 macrophages promote metastasis, survival, and tumor growth [122]. Clinically, macrophage polarization is correlated with the cancer stage. In the early stages of tumor inflammation, the TME attracts and promotes more macrophages to the pro-inflammatory M1 phenotype. Immunosuppressive M2 macrophages dominate in advanced tumor stages [125], although a mixed macrophage phenotype is also present [5].

Tumor-associated macrophages (TAMs) are innate immune cells composed of M2 macrophages with a small proportion of M1 macrophages [126]. Macrophages are attracted to chemokines and growth factors secreted from cancer cells; they infiltrate solid tumors and shift to the pro-tumor M2 subtype [125]. TAMs are the most abundant immune cells in the TME of solid tumors and play a critical role in tumor growth. TAM infiltration is clinically linked to poor prognosis in many solid tumor types [125]. Researchers are interested in targeting TAMs directly for therapeutic strategies because they are involved in many features of tumor progression, including cancer cell proliferation, tumor metastasis, and immune suppression [49]. Targeting TAMs has shown the potential to improve the efficacy of cancer therapies, but some limitations remain [49]. The complicated plasticity and heterogeneity of TAMs result in limited anti-tumor effects [48,127]. Additionally, the failure of M2-specific targets limits efficacy and results in a high level of toxicity due to the off-target impacts [48,127]. To optimize these complicated macrophage-based cancer therapies, researchers have found that adenoviral vector-based CAR can overcome the innate resistance of primary human macrophages to genetic engineering to generate CAR-M [121]. CAR-M demonstrated phagocytosis-based anti-tumor activity in vitro, boosted in vivo tumor-killing activity, and prolonged the overall survival rate in solid tumor xenograft mouse models and humanized mouse models [121]. Active CAR-M overcomes the solid tumor immunosuppressive TME by secreting pro-inflammatory cytokines and chemokines and upregulating the antigen presentation machinery for presenting antigens to T cells [128]. Table 6 summarizes the advantages, limitations, and strategies for improving CAR-M efficacy in solid tumor treatment. As described earlier, CAR-M has phagocytic activity against solid tumors—similar to M1 macrophages in the TME [121,122,125]. CAR-M can be generated from different sources. Human THP-1 cell lines, primary human macrophages derived from PBMC and iPSCs, can be engineered to express CAR [129]. Similar to CAR-T, the core structure of CAR-M is composed of an extracellular antigen recognition domain called scFv, a transmembrane domain, and an intracellular domain that activates downstream signaling pathways [129]. The cytotoxicity of CAR-M is activated by antigen-expressing tumor cells, and TME is modified by the activation of CAR-M [130]. M0 state CAR-M switches into an M1 pro-inflammatory phenotype and presents an anti-tumor effect when exposed to tumor antigen [121]. This activated CAR-M secretes pro-inflammatory cytokines and activates innate immune cells in TME. The phagocytic activity of CAR-M specifically recognizes and kills tumor cells [121]. CAR-Ms can also further enhance the adaptive immune system to induce synergistic anti-tumor effects by antigen presentation and activating the cytotoxicity of T cells [121]. The limited efficiency of CAR transduction by macrophages can be overcome using an adenoviral vector [121] and a Vpx-modified lentivirus [131]. The requirement of the M1 phenotype to produce CAR-M can be solved using iPSC [132], THP-1 macrophage cell lines [133], and PBMC [134].

Additionally, we summarize the relative advantages and disadvantages of CAR-NK versus CAR-M in Table 7 and Table 8. CAR-NK cells do not need complicated differentiation steps to have an M1 phenotype, and two mechanisms (CAR-dependent and -independent ADCC) regulate the anti-tumor activity [99] (Table 7). However, CAR-NK cells' tumor infiltration and persistence in solid tumor TME are very limited [104] (Table 7).

CAR-M cells have strong anti-tumor activity with phagocytosis of M1 macrophages infiltrated in TME of solid tumors [121] (Table 8). However, a differentiated M1 phenotype is required to engineer CAR-M cells [133] (Table 8).

### 4.2. Clinical Trials of CAR-M Therapies in Solid Tumors

Clinical trials of CAR-M cells in patients with solid cancers are presented in Table 9 [40]. NCT04660929 assessed the safety, tolerability, and feasibility of anti-HER2 CAR macrophages (CT-0508) [40]. This phase 1, open-label trial investigates the efficacy of adenovirally transduced autologous anti-HER2 CAR macrophages alone or in combination with pembrolizumab in HER2-overexpressing solid tumors [40]. A pre-clinical study demonstrated that CAR-M showed antigen-specific phagocytosis and reduced tumors in vitro [121]. Additionally, CAR-M decreased the tumor size and volume and increased the overall survival rate in solid tumor xenograft mouse models [121]. Moreover, CAR-M enhanced the anti-tumor activity of T cells in the pro-inflammatory TME [121]. In September 2021, the US FDA granted the Fast Track designation to CT-0508 to facilitate review and frequent communication with the FDA [135].

NCT05007379 determined the efficacy of new CAR macrophages against organoids derived from early and advanced breast cancer patients [40]. Organoids from HER2-negative, low, and positive breast cancers were examined in this trial [40]. Moreover, the anti-tumor activity of non-modified macrophages was compared with that of new CAR macrophages [40].

## 5. Conclusions

Although CAR-T cell therapy has shown excellent clinical success in hematological malignancies, many challenges and obstacles remain. CRS, ICANS, immune escape, limited tolerance to the TME, high relapse rate, and high treatment costs are representative problems of CAR-T therapies that need to be overcome. These limitations of CAR-T cells have led to other immune cells, such as NK cells and macrophages, becoming potential alternatives. CAR-NK cells have the characteristics of potential "off-the-shelf" treatments with less cytotoxicity, lower costs, and a shorter lifespan than CAR-T cells. CAR-M can overcome the TME and better control solid tumors; therefore, they can be applied to a broad spectrum of patients with solid tumors. These features allow CAR-NK and CAR-M cells to be regarded as favorable alternatives to CAR-T cells to overcome these limitations and achieve safe and efficient therapeutic applications for cancer treatment. Further in-depth investigations to develop novel CAR technology with NK cells, macrophages, and T cells and to identify innovative combination therapeutic strategies should be conducted to overcome the present restrictions.

## Figures and Tables

**Table 1:** Advantages, limitations, and strategies to improve CAR-T cell therapies.

**Advantages**	Short treatment time—generally requires a single infusion [6] Prolonged durability [7,8] Strong efficiency in hematologic malignancies [9,10,11]
**Limitations ** **-Strategies**	Lack of antigen specificity and heterogeneity - CAR-T cells targeting two or more antigens [13,14] - Induction of bystander effects by CAR-T cells [15,16] Difficulty in tumor trafficking and infiltration - CAR-T cells expressing chemokine receptors [17] - CAR-T cells targeting stromal cell-associated antigens of TME [21] Limited persistence in the immunosuppressive solid tumor microenvironment (TME) - CAR-T cells expressing immune cell activating cytokines (e.g., IL-12, IL-18, and IL-15) [25,26] - CAR-T cells targeting immunosuppressive cells (e.g., M2 macrophages, MDSCs, and Tregs) [27] - CAR-T cells in combination with checkpoint inhibitors [28] - CAR-T cells expressing PD-1 and/or CTLA-4 monoclonal antibodies [23] Toxicities: cytokine release syndrome (CRS) and immune effector cell-associated neurotoxicity syndrome (ICANS) - IL-6 blockade [29] - Improvement of CAR design (e.g., suicide gene incorporation) [30] GvHD - Development of "off-the-shelf" allogenic CAR-T cells [31]

**Table 2:** FDA-approved CAR-T cell therapies.

Brand Name	Generic Name	Target Antigen	Indications	Manufacturer	Date of FDA Approval
Kymriah	Tisagenlecleucel	CD19	Relapsed or refractory large B-cell lymphoma (LBCL) Relapsed or refractory B-cell acute lymphoblastic leukemia (ALL) Relapsed or refractory follicular lymphoma (FL)	Novartis Pharmaceuticals Corporation (Basel, Switzerland)	30 August 2017
Yescarta	Axicabtagene ciloleucel	CD19	Relapsed or refractory large B-cell lymphoma (LBCL) Relapsed or refractory follicular lymphoma (FL)	Kite Pharma, Inc. (Los Angeles, CA, USA)	18 October 2017
Tecartus	Brexucabtagene autoleucel	CD19	Relapsed or refractory mantle cell lymphoma (MCL) Relapsed or refractory B-cell acute lymphoblastic leukemia (ALL)	Kite Pharma, Inc.	24 July 2020
Breyanzi	Lisocabtagene maraleucel	CD19	Relapsed or refractory large B cell lymphoma (LBCL) Relapsed or refractory follicular lymphoma (FL)	Juno Therapeutics, Inc., (Seattle, WA, USA) Bristol-Myers Squibb Company (New York, NY, USA)	5 February 2021
Abecma	Idecabtagene vicleucel	BCMA	Relapsed or refractory multiple myeloma (MM)	Celgene Corporation, (Summit, NJ, USA) Bristol-Myers Squibb Company	26 March 2021
Carvykti	Ciltacabtagene autoleucel	BCMA	Relapsed or refractory multiple myeloma (MM)	Janssen Biotech, Inc. (Horsham, PA, USA)	28 February 2022

**Table 3:** Representative clinical trials of CAR-T cell therapies in patients with solid tumors.

National Clinical Trial (NCT) Number	Title	Status	Conditions	CAR-T Product	Targeted Antigen	Modification to Overcome the Limitations	Phase	Start Date
NCT 05693844	CD40 ligand-expressing MSLN CAR-T cell therapy in MSLN-positive advanced/metastatic solid tumors	Recruiting	Advanced or metastatic solid tumors	CD40 ligand expressing MSLN-CAR-T cells	MSLN	Targeting both CD40 and MSLN	Phase 1 Phase 2	20 January 2023
NCT 05621486	A clinical study to evaluate B4T2-001 CAR-T cells in the treatment of advanced solid tumors	Recruiting	Advanced solid Tumor	B4T2-001 CAR-T cells	BT-001	Targeting novel-, self-antigen	Phase 1	14 September 2022
NCT 05518253	A clinical study of CD70-targeted CAR-T in the treatment of CD70-positive advanced/metastatic solid tumors	Recruiting	Metastatic tumor, Advanced solid tumor, Renal cell carcinoma, Ovarian cancer, Cervix cancer	CD70 CAR-T cells	CD70	Administratio method: Intravenous infusion versus Intraperitoneal injection	Phase 1	30 May 2022
NCT 05120271	BOXR1030 T cells in subjects with advanced GPC3-positive solid tumors	Recruiting	Hepatocellular carcinoma, Squamous cell carcinoma of the lung, Merkel cell carcinoma, Myxoid/round cell liposarcoma	GPC3-CAR-T cells	GPC3	Expressing exogenous GOT2 (Glutamic-oxaloacetic transaminase 2) for optimal T cell activity	Phase 1 Phase 2	18 May 2022
NCT 05382377	NKG2D CAR-T(KD-025) in the treatment of advanced NKG2DL+ solid tumors	Recruiting	CRC, Solid tumor	NKG2D CAR-T cells (KD-025)	NKG2DL	Potent anti-tumor activity with upregula- ting TNFa, IFN-γ, IL-10 and IL-2 cytokines	Early Phase 1	17 May 2022
NCT 05239143	P-MUC1C-ALLO1 allogeneic CAR-T cells in the treatment of subjects with advanced or metastatic solid tumors	Recruiting	Breast cancer, Ovarian cancer, Non-small cell lung cancer, Colorectal cancer, Pancreatic cancer, Renal cell carcinoma, Nasopharyngeal cancer, Head and neck squamous cell carcinoma, Gastric cancer	P-MUC1C-ALLO1 CAR-T cells	MUC1-C	Allogeneic CAR-T cells	Phase 1	15 February 2022
NCT 05199519	Study to Evaluate the Safety, Tolerance, Pharmacokinetics and Preliminary Efficacy of IBI345	Completed	CLDN18.2 Positive Solid Tumors	IBI345 (IBI345 CAR-T cell)	CLDN18.2	Allogeneic CAR-T cells	Phase 1	13 December 2021
NCT 05103631	Interleukin-15 armored Glypican 3-specific chimeric antigen receptor expressed in autologous T cells for hepatocellular carcinoma	Recruiting	Liver cell carcinoma, Solid tumor, Wilms tumor, Malignant rhabdoid tumor, Yolk sac tumor, Rhabdomyosarcoma, Liposarcoma, Embryonal sarcoma of the liver	Interleukin-15 armored Glypican 3-specific CAR-T cells	GPC3	Engineered to express IL-15 to enhance anti-tumor activity	Phase 1	17 June 2021
NCT 04581473	A study to evaluate the efficacy, safety, and pharmacokinetics of CT041 autologous CAR-T cell injection	Recruiting	Gastric adenocarcinoma Pancreatic cancer, Gastroesophageal junction adenocarcinoma	CT041 CAR-T cells	CLDN18.2	CLDN18.2, a digestive system cancer-specific biomarker	Phase 1 Phase 2	23 October 2020
NCT 04511871	A phase I trial of CCT303-406 in patients with relapsed or refractory HER2-positive solid tumors	Recruiting	Solid tumor, Gastric cancer, Breast cancer, Ovarian cancer, Sarcoma	HER2 CAR-T cells	HER2	Requiring both target antigen and TME to activate CAR-T cells	Phase 1	9 July 2020
NCT 03170141	Immunogene-modified T (IgT) Cells Against Glioblastoma Multiforme	Enrolling by invitation	Glioblastoma Multiforme of Brain, Glioblastoma Multiforme	AutologousGD2-specific fourth-generation safety-designed chimeric antigen receptor (4SCAR)-T cells	GD2	Contains a suicide gene safety switch (namely inducible Caspase 9)	Phase 1	31 May 2020
NCT 04348643	Safety and efficacy of CEA-targeted CAR-T therapy for relapsed/refractory CEA+ cancer	Recruiting	Solid tumor, Lung cancer, Colorectal cancer, Liver cancer, Pancreatic cancer, Gastric cancer, Breast cancer	CEA CAR-T cells	CEA	CEA, a digestive tract cancer-specific biomarker	Phase 1 Phase 2	20 February 2020
NCT 05812326	PD-1 Knockout Anti-MUC1 CAR-T Cells in the Treatment of Advanced Breast Cancer	Completed	Advanced Breast Cancer, Breast Neoplasm Malignant Female	AJMUC1- PD-1 gene knockout anti- MUC1 CAR-T cells	MUC1	PD-1 knockout	Phase 1 Phase 2	17 May 2019
NCT 03373097	Anti-GD2 CAR-T cells in pediatric patients affected by high-risk and/or relapsed/refractory neuroblastoma or other GD2-positive solid tumors	Recruiting	Neuroblastoma, Recurrent neuroblastoma, GD2-positive solid tumors, Osteosarcoma, Ewing sarcoma, Sarcoma	GD2 CAR-T cells	GD2	Contains a suicide gene safety switch (namely inducible Caspase 9)	Phase 1 Phase 2	5 January 2018
NCT 03198546	GPC3-CAR-T Cells for Immunotherapy of Cancer With GPC3 Expression	Recruiting	Hepatocellular Carcinoma Immunotherapy, CAR GPC3 Gene Inactivation, T Cell, Squamous Cell, Lung Cancer	GPC3 targeting CAR-T cells	GPC3	Engineered to secrete human IL-7 and CCL19 for enhanced anti-tumor activity	Phase 1	1 July 2017
NCT 03851146	A Study of Anti-Lewis Y Chimeric Antigen Receptor-T Cells (LeY-CAR-T) in Patients With Solid Tumours (LeY-CAR-T)	Completed	Advanced cancer	LeY CAR T cells	LeY (Lewis Y)	High levels of IFN-γ, TNFα and IL-2 secretion following direct stimulation through LeY+ tumor targets.	Phase 1	24 November 2016
NCT 02761915	A Phase I Trial of Anti-GD2 T-cells (1RG-CART)	Completed	Relapsed or Refractory Neuroblastoma	1RG-CART	GD2	RQR8 suicide gene is incorporated	Phase 1	29 February 2016

**Table 4:** Advantages, limitations, and strategies to improve CAR-NK cell therapies.

**Advantages**	Can be generated from different sources (peripheral blood, cord blood, iPSCs, and cell lines) [99,100,101,102,103] Anti-tumor activity is derived from both CAR-dependent and -independent ADCC mechanisms [99] Low risk of GvHD, CRS, and ICANS [99] “off-the-shelf” allogeneic CAR-NK cell therapy [99]
**Limitations** **-Strategies**	Lack of tumor infiltration - Modified CAR-NK cells to express chemokine receptors [41] - CAR-NK cells designed to target Tregs, mesenchymal stromal cells, and cancer-associated fibroblasts [104] Low CAR transduction efficiency - Non-viral electroporation (mRNA, transposon) [105,106] - Small molecular compounds to enhance the viral transduction of NK cells (e.g., retronectin, vectofusin-1, and PEG2) [106] Limited ex vivo expansion - Engineered feeder cells to express immunostimulatory signaling molecules (e.g., IL-21, IL-15, and 4-1BB) [109,110] - Engineered CAR-NK cells to express IL-12, IL-15, and IL-18 [104,111,112] Limited persistence in the immunosuppressive TME - CAR-NK cells expressing immune cell-stimulating cytokines (e.g., IL-12, IL-18, and IL-15) [41,106] - CAR-NK cells in combination with immune checkpoints inhibitors [41,104]

**Table 5:** Clinical trials of CAR-NK cell therapies in patients with solid tumors.

National Clinical Trial (NCT) Number	Title	Status	Conditions	CAR-NK Product	Targeted Antigen	Modification to Overcome the Limitations	Phase	Start Date
NCT 05528341	NKG2D-CAR-NK92 cell immunotherapy for solid tumors	Recruiting	Relapsed/refractory solid tumors	NKG2D CAR-NK92 cells	NKG2DL	Off-the-shelf NK92 cell line-based CAR-NK	Phase 1	26 January 2023
NCT 05410717	CLDN6-CAR-NK cell therapy for advanced solid tumors	Recruiting	Stage IV ovarian cancer, Testicular cancer, refractory Endometrial cancer, recurrent	Claudin6 targeting CAR-NK cells	Claudin6	Engineered to express IL7/CCL19 and/or scfv against PD1/CTLA4/Lag3	Phase 1 Phase 2	1 June 2022
NCT 05194709	Study of anti-5T4 CAR-NK cell therapy in advanced solid tumors	Recruiting	Advanced solid tumors	Anti-5T4 CAR-NK Cells	Oncofetal trophoblast glycoprotein (5T4)	Targeting 5T4 (oncofetal antigen) which allow survival of tumor in its host	Early Phase 1	30 December 2021
NCT 05137275	Study of anti-5T4 CARraNK cell therapy in locally advanced or metastatic solid tumors	Recruiting	Locally advanced or metastatic solid tumors	Anti-5T4 CAR-raNK (allogeneic NK) Cells	Oncofetal trophoblast glycoprotein (5T4)	Allogeneic CAR-NK cells	Early Phase 1	24 November 2021
NCT 03940820	Clinical Research of ROBO1 Specific CAR-NK Cells on Patients With Solid Tumors	Unknown	Solid tumor-Pancreatic cancer	ROBO1 CAR-NK cells	ROBO1 (Roundabout homolog 1)	Off-the-shelf NK92 cell line-based CAR-NK	Phase 1 Phase 2	May 2019
NCT 03415100	Pilot Study of NKG2D-Ligand Targeted CAR-NK Cells in Patients With Metastatic Solid Tumours	Unknown	Metastatic solid tumors	CAR-NK cells targeting NKG2D ligands	NKG2DL	Allogeneic CAR-NK cells	Phase 1	2 January 2018
NCT 02839954	CAR-pNK Cell Immunotherapy in MUC1 Positive Relapsed or Refractory Solid Tumor	Unknown	Hepatocellular Carcinoma, Non-small Cell Lung Cancer, Pancreatic Carcinoma, Triple-Negative Invasive Breast Carcinoma, Malignant Glioma of Brain, Colorectal Carcinoma, Gastric Carcinoma	anti-MUC1 CAR-pNK cells	MUC1 (Mucin 1)	Targeting MUC1 (Mucin 1) on epithelial surfaces for enhanced tumor infiltration	Phase 1 Phase 2	July 2016

**Table 6:** Advantages, limitations, and strategies to improve CAR-M therapies.

**Advantages**	Pro-inflammatory and anti-tumor activity by phagocytosis of M1 macrophages in TME [121,122,125] Various sources (cell lines, PBMC, iPSC) [130] Macrophages are abundant in the TME of solid tumors [121].
**Limitations** **-Strategies**	CAR transduction efficiency is restricted - Chimeric adenoviral vector [121] - Vpx-modified lentivirus [131] Differentiation into M1 phenotypes is required - iPSC-derived CAR-macrophages [132] - Differentiation of human THP-1 macrophage cell lines into M1 phenotypes [133] - M1 derivation from human PBMC [134]

**Table 7:** Relative advantages and disadvantages of CAR-NK versus CAR-M therapies.

**Advantages**	Differentiation into M1 phenotypes is not required [99] Both CAR-dependent and -independent ADCC mechanisms are involved in anti-tumor activity [99]
**Disadvantages**	Limited infiltration into TME of solid tumors [104] Limited persistence in the immunosuppressive TME [104]

**Table 8:** Relative advantages and disadvantages of CAR-M versus CAR-NK therapies.

**Advantages**	Pro-inflammatory and anti-tumor activity by phagocytosis of M1 macrophages in TME [121] Macrophages are abundant in the TME of solid tumors [121]
**Disadvantage**	Differentiation into M1 phenotypes is required [133]

**Table 9:** Clinical trials of CAR M therapies in patients with solid tumors.

National Clinical Trial (NCT) Number	Title	Status	Conditions	CAR-T Product	Targeted Antigen	Modification to Overcome the Limitations	Phase	Start Date
NCT 04660929	CAR-macrophages for the treatment of HER2-overexpressing solid tumors	Recruiting	HER2-positive Adenocarcinoma, Bile duct cancer, Biliary tract cancer, Bladder cancer, Breast cancer, Breast neoplasm, Carcinoma, ductal Carcinoma, hepatocellular and 21 more	Anti-HER2 CAR macrophages	HER2	Adenoviral vector CAR, Combination treatment wirh pembrolizumab	Phase 1	2 February 2021
NCT 05007379	Cohort study to determine the anti-tumor activity of new CAR macrophages in the derived organoids of breast cancer patients	Not yet recruiting	Breast cancer	Anti-HER2 CAR macrophages	HER2	CAR-M efficacy testing against patient-derived organoids		1 September 2021

## Data Availability

No new data were created or analyzed in this study. Data sharing is not applicable to this article.
